# Standardization of sample collection, isolation and analysis methods in extracellular vesicle research

**DOI:** 10.3402/jev.v2i0.20360

**Published:** 2013-05-27

**Authors:** Kenneth W. Witwer, Edit I. Buzás, Lynne T. Bemis, Adriana Bora, Cecilia Lässer, Jan Lötvall, Esther N. Nolte-‘t Hoen, Melissa G. Piper, Sarada Sivaraman, Johan Skog, Clotilde Théry, Marca H. Wauben, Fred Hochberg

**Affiliations:** 1 Department of Molecular and Comparative Pathobiology, Johns Hopkins University School of Medicine, MD, USA; 2 Department of Genetics, Cell- and Immunobiology, Semmelweis University, Budapest, Hungary; 3 Department of Biomedical Sciences, University of Minnesota Medical School Duluth, Duluth, MN, USA; 4 Department of Pharmacology and Molecular Sciences, Johns Hopkins University School of Medicine, Baltimore, MD, USA; 5 Krefting Research Centre, Department of Internal Medicine and Clinical Nutrition, Sahlgrenska Academy, University of Gothenburg, Gothenburg, Sweden; 6 Department of Biochemistry and Cell Biology, Faculty of Veterinary Medicine, Utrecht University, Utrecht, The Netherlands; 7 Department of Internal Medicine, College of Medicine, The Ohio State University, Columbus, OH, USA; 8 Massachusetts General Hospital Cancer Center, Boston, MA, USA; 9 Exosome Diagnostics, Inc., New York, USA; 10 INSERM, Cedex, Paris, France; 11 Institut Curie, Paris, France

**Keywords:** extracellular vesicle, exosome, microvesicle, standardization, isolation

## Abstract

The emergence of publications on extracellular RNA (exRNA) and extracellular vesicles (EV) has highlighted the potential of these molecules and vehicles as biomarkers of disease and therapeutic targets. These findings have created a paradigm shift, most prominently in the field of oncology, prompting expanded interest in the field and dedication of funds for EV research. At the same time, understanding of EV subtypes, biogenesis, cargo and mechanisms of shuttling remains incomplete. The techniques that can be harnessed to address the many gaps in our current knowledge were the subject of a special workshop of the International Society for Extracellular Vesicles (ISEV) in New York City in October 2012. As part of the “ISEV Research Seminar: Analysis and Function of RNA in Extracellular Vesicles (evRNA)”, 6 round-table discussions were held to provide an evidence-based framework for isolation and analysis of EV, purification and analysis of associated RNA molecules, and molecular engineering of EV for therapeutic intervention. This article arises from the discussion of EV isolation and analysis at that meeting. The conclusions of the round table are supplemented with a review of published materials and our experience. Controversies and outstanding questions are identified that may inform future research and funding priorities. While we emphasize the need for standardization of specimen handling, appropriate normative controls, and isolation and analysis techniques to facilitate comparison of results, we also recognize that continual development and evaluation of techniques will be necessary as new knowledge is amassed. On many points, consensus has not yet been achieved and must be built through the reporting of well-controlled experiments.

Extracellular vesicles (EV) have garnered considerable attention because of the specific mechanisms whereby they are actively released from cells; their involvement in cell-to-cell signalling; and their utility as markers of disease (1–12). Extracellular RNA (exRNA) is carried and protected within the extracellular milieu by EV and other vehicles, such as protein complexes (13, 14) and lipoprotein particles (15, 16). This RNA includes microRNA (miRNA), transfer RNA (tRNA), ribosomal RNA (rRNA), messenger RNA (mRNA), and additional structural and non-coding RNAs (17, 18). Although some RNAs may be incorporated non-specifically, there is growing evidence for enrichment of certain RNA species in specific vesicle types (1, 17, 18). Along with other EV-associated molecules, specific EV exRNAs may participate in signalling processes and serve as molecular signatures of disease.

## Nomenclature

EV are a heterogeneous group, and the nomenclature is still being defined and refined by the research community (19–21). This process will likely continue for the foreseeable future because of the many outstanding questions associated with biogenesis, uptake and other processes (22). For example, “exosomes” have classically been defined as originating from the endosomal compartment by fusion of multivesicular bodies (MVB) with the plasma membrane, whereas “microvesicles”, “ectosomes” or “shed vesicles/particles” have been thought to originate by direct budding from the plasma membrane (16, 23). In the literature, exosomes are often described as smaller than 100 nm in diameter, while microvesicles are considered to be larger than 100 nm (7, 16, 24). However, the strict separation of these vesicle types by size or biogenesis has not been established beyond doubt (25–27), and there is currently no consensus on markers that distinguish the origin of these vesicles once they have left the cell (25). As numerous participants in the ISEV workshop noted, there is little evidence that particles less than 100 nm in diameter cannot bud from the plasma membrane, and EV with exosome markers may be larger than 100 nm in size. In terms of biogenesis and markers, retroviral particles are in reality a type of EV that may be exemplary of a blurring of identity of the classic “exosome” and “microvesicle” (25, 27–30). In using the term EV to describe all classes of extracellular membrane vesicles, we recognize that the future will likely bring increased specificity of terms used in this emerging field and that consensus does not yet exist.

## Biofluids

EV are present in biological fluids (31, 32). At least some EV are associated with exRNA molecules, in which case the RNA may be referred to as evRNA. EV have been isolated from biofluids including pleural effusions (33), plasma (34, 35), ocular effluent and aqueous humor (36), breast milk (35, 37), ascites (38, 39), amniotic fluid (40, 41), semen (“prostasomes” and “epididymosomes”) (42–50), saliva (35, 41, 51, 52), nasal secretions (53), cerebrospinal fluid (CSF) (54), bronchoalveolar lavage (BAL) (55–57), synovial fluid (58–65), bile (66) and urine (67, 68). EV are commonly recovered from cell culture-conditioned media (69–71). Vesicles have also been recovered from dissociated tissue [e.g. adipose tissue (72) and thymus (73)]; however, it is difficult to establish the extracellular nature of these vesicles, as the recovered materials can also comprise intracellular vesicles released during the tissue dissociation process.

EV in biological fluids – and the molecules associated with them, including RNA – may be used diagnostically for different disease states. Accordingly, they have aroused considerable interest in the research community and studies have been initiated for biomarker discoveries in diseases such as cancers of the urogenital system (bladder, prostate and ureter), melanoma, and ovarian and primary brain tumours. These consortia are based on early explorations of the utility of EV RNA of prostate-specific antigen (PSA), prostate cancer antigen 3 (PCA3), and the gene fusion of transmembrane protease, serine 2 and Ets-related gene (TMPRSS2: ERG) in the urine of prostate cancer patients (74); the protein claudin-4 in blood EV in ovarian cancer (75); and the epidermal growth factor receptor variant EGFRvIII in gliomas (76). Notable is a multi-institution consortium collaborating to validate the glioblastoma diagnostic CSF biomarker EGFRvIII as well as isocitrate dehydrogenase with a codon 132 mutation (IDH1.132) (76). In parallel with these biomarker trials, the utility of EV isolates for vaccination against cancer includes vaccination trials for melanoma (77) and non-small-cell lung carcinoma (78) using EV from patients’ dendritic cells loaded with tumour antigen-derived peptides, and colorectal cancer with EV from colon cancer patients’ ascites combined with granulocyte-macrophage colony-stimulating factor [GM-CSF (38)]. In addition, a vaccination trial has utilized a group B meningococcal bacterial outer membrane vesicle with similarities to mammalian plasma membrane-derived vesicles (79, 80).

## Need for standardization

In the midst of growing interest in EV, technical standardization is of central importance because many methodologies have been used to isolate and analyze EV. Following isolation, a variety of techniques have been employed to purify RNA. The influence of these disparate techniques on the results of downstream exRNA sequencing and profiling remains unclear, raising the need to provide a definition of “best practices” and eventual standardization. As exRNA diagnostic platforms become available, there will be requirements for clinical certification and development of manufacturing standards. Furthermore, pressure will emerge to define and centralize biofluids from normative biological controls.

## ISEV 2012 workshop on evRNA

To host discussion and foster progress towards resolving the technical issues of evRNA research, the International Society for Extracellular Vesicles (ISEV) organized a meeting in New York City. Under the leadership of ISEV president Jan Lötvall and Board Member Marca Wauben, the “ISEV Research Seminar: Analysis and Function of RNA in Extracellular Vesicles (evRNA)” was convened on 1–2 October 2012 (81). Following on from the Society's first annual meeting in the spring of 2012 (82), this workshop was attended by an international and experientially diverse group of participants from academia and industry. The format included short presentations as well as 6 round table discussions (Table I).

**Table I:** 2012 ISEV Research Seminar discussion topics

Round table	Topic	Description
1	EV isolation	Pelleting, filtration, density gradient, controls
2	RNA isolation	Comparison of different isolation protocols
3	evRNA analysis	Hybridization, qPCR, RNase treatment
4	evRNA analysis and bioinformatics	Deep sequencing, analysis pipelines, recent developments
5	RNA loading and engineered vesicles	Electroporation, loading control, cellular production, targeting
6	Analysis of functional evRNA transfer	Reporter systems, physiological read-out systems

In this article, we summarize the outcome of the ISEV workshop discussions and subsequent communications pertaining to pre-analytical variables in EV research (chiefly derived from topic 1, Table I). We present study design considerations, including planning for appropriate biological normatives, to maximize the utility of EV research projects. Second, we focus on plasma as an example through which to review important considerations in sample collection procedures and processing variables, followed by presentation of challenges specific to a variety of additional biological fluids. We evaluate EV isolation methods and critique quantitative and qualitative methods for EV analysis. Finally, we summarize several suggestions (see also Table II) to guide progress in the field.

**Table II:** Recommendations: resource development

Resource	Rationale	Details	Resource availability
Centralized normative control biofluid bank	For rigorous comparison of EV isolation methods, inter-study comparisons, and well-controlled biomarker development	Well-characterized normative matched biofluid samples (e.g. blood, CSF, urine, saliva) with “full disclosure” medical information and other details	Distribution by NIH or other repository; researchers would submit project-specific proposals for access
Artificial EV spike-in standards	To standardize recovery of EV and evRNA from biofluids; to facilitate independent verification of results	Four or five distinct vesicle sizes, closely mimicking bioparticles; loaded or not with markers (potentially inducible?): fluorescent protein or specific RNA sequences	Commercial or distributed by a repository to consortium members; established laboratories would be available for validation studies
Additional funding mechanisms for technique and normative control development	Disparity of results is dependent in part on procedural differences that could be resolved	Examples: head-to-head EV isolation comparisons; reproducibility of EV recovery throughout the day; sample volume and recovery studies; methods for determination of cellular/tissue origin of circulating EV	Standard grant application procedures
Collaborative sample and resource sharing network	Many groups focus on 1 particular type or size of EV or RNA; studies of different EV and evRNAs in the same samples would be useful	Participants could analyze different aliquots of the same biological fluids or send EV/fluids for analysis by laboratories with different focus/expertise	Website and voluntary cooperation

## Study design and normative controls

### The goal of EV research projects will influence isolation procedures

EV research can be broadly divided into 3 categories, each of which mandates a different level of quality control and operating procedure. Since many technical protocols exist, some more closely suited than others to particular types of research, it is necessary to select protocols based on the research question.

- *Discovery research* characterizes EV quantity, size, exRNA cargo, proteomics, lipidomics, metabolomics, glycomics and other characteristics in body fluids. To discover candidate biomarkers in general EV populations or those sequestered by size, structure or charge, attention must be paid to reproducibility of the assay, minimization of “non-target” EV (e.g. in some but certainly not all approaches, the broad background of platelet- and neutrophil-derived vesicles) and optimization of assay sensitivity. Thus, isolation protocols are important, but function of isolated EV does not necessarily need to be preserved.
- *Diagnostic research* involves the search for features that are uniquely or quantitatively associated with a disease process. In order to validate the clinical specificity or correlation strength of a marker, selective EV isolation may be imperative, depending on the biofluid. A clinically useful test should offer advantages of increased sensitivity, reduced cost or lowered morbidity when compared to current diagnostic methods when evaluated in a selected patient population and when compared with appropriate population controls.
- *Preparative research* involves the preparation of EV to be given to patients (43) or for mechanistic analyses. This research requires the highest level of standards aimed both to preserve EV function and to selectively purify EV.

### Normative controls

There is a need to define biofluid-specific normatives for EV studies as well as standardized methods, research designs and “control groups” that mirror demographics of patients except for disease status. For patients, controls could be *relatives accompanying the patient* (in particular if they are sex- and/or age-matched individuals), an approach common to epidemiologic case-control studies.

Many factors have known or suspected influence on vesiculation. These include *age, gender, body mass index (BMI) and medication use (e.g. oral contraceptives)*. As yet, no systemic studies have investigated the effect of *race- or ethnicity-associated polymorphisms* on vesiculation, but, if feasible, it may be advisable to recruit race-matched controls for the investigations. Among women, controls should be selected for *pregnant, non-pregnant* and *multiple pregnancy samples* (83). *Fasting or postprandial status* may affect blood plasma lipoprotein and EV levels (84). *Current sickness*, including common cold symptoms, should be assessed since acute infection alters circulating cells and RNA (85, 86). *Hospitalization* and *physical activity* (e.g. regular training or exercise prior to sampling) should be matched in test and control groups (84, 87–89). Individuals on *heparin therapy* should be given special attention because of the interference of heparin with reverse transcription and PCR (9) and with EV/recipient cell interaction (90).

*Repeated samples* from patients may serve as controls not skewed by individual differences (86). Aliquots can be used to validate EV isolation, RNA extraction and assay variability, whereas samples from the same individual drawn at multiple time points can be used for variability testing of a particular EV marker. Emphasis should be placed on matching samples from controls with samples from patients obtained prior to surgical disease diagnosis of cancer or cultured proof of infection. Matched intra-individual pre-disease or pre-infection samples would provide the ultimate baseline for personalized assays. Establishment of pre-disease sample banks for individuals within a healthcare system should be considered carefully, but widespread implementation is unlikely in the near future. In the absence of ideal matched controls, normative samples should be chosen carefully.

### Maximizing sample utility in harmony with local and international ethical and legal standards

The collection and use of samples from human subjects are governed by complex systems of regulations and guidelines that often differ by country or other geopolitical entities and even from institution to institution. For example, the Declaration of Helsinki of the World Medical Association and the Good Clinical Practice standard of the International Conference on Harmonisation aim to protect the rights of individuals in medical research, while privacy regulations have been enacted in various locations to control the availability of patient-specific medical information. To maximize the utility of each sample, it may be important to draft applications with the broadest possible language to be compatible with all conceivable experimental and information requirements. International efforts are also needed to construct identified and normative donor pools matched to patient demographics to be studied in harmony with international standards. For example, in George Church's Personal Genome Project (http://www.personalgenomes.org/), donors volunteer personal genomic materials and personal information (91–93).

### Sample size determination

Reliance on sound statistics is needed when estimating population sizes by which to “power” study design calculations. That this seemingly obvious consideration bears emphasis is evident from a recent systematic review of miRNA microarray publications (not restricted to EV studies) (94). Almost 40% of reviewed studies drew conclusions from a sample size of just one in at least one experimental group, eliminating the possibility of meaningful statistical analysis (94). Of course, as next-generation sequencing and other profiling techniques can be prohibitively expensive, consortia should be cultivated to achieve larger group sizes.

### Database submission

Data submission is mandated by most journals (95, 96) and independent studies have found that unavailability of data is associated with low data or study quality (94, 97, 98); however, compliance remains poor (94, 99, 100). Investigators should plan to provide to a public database (101–103) complete nucleic acid sequencing and profiling data, along with detailed technical and analytical methods. Furthermore, authors are encouraged to submit EV-related molecular data (RNA, protein and lipid) to EV-specific databases Vesiclepedia [104; http://microvesicles.org)] and EVpedia [105; http://evpedia.info/]. Vesiclepedia is a compendium for EV data with continuous community annotation and was created through the efforts of investigators associated with the ISEV. Founding members of the Vesiclepedia have agreed to deposit their data to make it publically accessible.

## Sample collection and processing

We begin our review of sample collection and processing considerations by examining the major biofluid, blood. A checklist of information that should be collected is provided as Box 1. Considerations specific to several selected additional biological fluids and tissue culture-conditioned media – each of which has its own compositional complexities – are then provided.

***Box 1*. A proposed checklist for sample collection and processing**

**Background information**

Age

Sex

Race/ethnicity

Females: any current pregnancy, gravidity, ovulatory cycle and menopause state

Hospitalization (recent)

Prior or current anticoagulant treatment or coagulopathy

  Heparin therapy

  Heparinoids

  Aspirin

  Known clotting disorder of peripheral veins, lung   (pulmonary embolus)

Current medication list (Including but not limited to birth control)

Smoker?

Drugs of abuse?

Physical activity-each week

  Daily job or home activities

  Dedicated physical activity <3 h per week

  Physical activity >3 h per week

Sleep pattern (e.g. night shift worker/sleep disturbances)

Last food and fluid intake

  Additional dietary details if possible

Sample collection and processing (blood)

Time sample obtained

Needle type/other apparatus used

Anticoagulant(s) and tube types

How many millilitres were discarded at initiation of draw? Was tourniquet removed promptly?

Time interval between draw and centrifugation

Notes on handling (tube kept upright? room temperature?)

Haemolysis and method of assessment

Sample processing details

  Rotor type

  rcf

  *k*-Factor

  time

Sample aliquot details and storage

Were cells obtained? Notes.

  Complete blood count?

  Flow cytometry panel (specify)?

Other assays performed on blood?

Were additional matched samples collected? (e.g. plasma and serum, urine, nasal secretions, saliva)

Were samples obtained from an accompanying friend or family member as a control (for patients)?

### Blood

#### Choice of plasma or serum

For most studies involving isolation of EV RNA, we encourage biobanking of plasma. Retrieved vesicles are consistently more abundant in sera than in plasma (106), and platelet-derived EV released *after* blood collection during the process of clot formation (107) may account for over 50% of EV in serum. Thus, although serum certainly has appropriate uses (108), plasma is the physiological medium of EV in the blood. In the majority of published studies on circulating EV, plasma (and not serum) samples were tested. Some participants in the 2012 ISEV workshop noted that, for some research questions, the use of serum – and even the presence of platelet-derived EV released after sample collection – might be desirable. For example, if platelets were to contain disease-specific mutations or nucleic acid profiles, platelet-derived EV might be diagnostic for some diseases, whereas total plasma exRNA might not be diagnostic (109). Since most comparisons of exRNA in plasma and serum have used whole plasma and serum (109–112), not RNA isolated from purified EV, we recommend that additional studies be undertaken to increase our understanding of plasma/serum differences and how they are affected by sample processing protocols.

#### Choice of anticoagulant

Anticoagulants have one or more modes of action, including calcium chelation, protease inhibition and inhibition of platelet activation. With many anticoagulants available, it is perhaps easiest to make a negative recommendation: use of heparin-based anticoagulants is discouraged. Whether of exogenous or endogenous origin (e.g. from mast cells), heparin is associated with false-negative PCR reads (113–115), presumably because heparin competes with primers and/or enzymes for binding to nucleic acid (116). In addition to PCR inhibition, heparin can bind EV, block EV uptake by cells (90), EV activate platelets (117) or lower the platelet activation threshold (118). Care should be taken to avoid intravascular “lines” with heparin or heparinoid infusions or those “cleared” using these materials, and the heparin treatment status of the patient should be recorded. In the case of valuable archived samples, it may be necessary to extract RNA from heparinized blood products. Detection of nucleic acids has been reported following heparinase treatment (119–121) or through the use of polymerases that are relatively insensitive to inhibitors (119). If EV-associated nucleic acids are found exclusively inside vesicles, extensive washing may also help to remove heparin prior to RNA isolation, but additional studies are needed.

The choice of an alternative anticoagulant – for example EDTA, sodium fluoride/potassium oxalate (NaF/KOx), or sodium citrate with or without additives such as dextrose (ACD) or theophylline, adenosine and dipyridamole (CTAD) – is complicated and might best be guided by the intended downstream assays. CTAD blocks activation of platelets and subsequent EV release (122, 123). EDTA may interfere with PCR reactions (albeit to a lesser extent than heparin), but its presence may be innocuous or even beneficial for other purposes. Thus, it may be useful to collect blood in separate tubes with different anticoagulants. Alternatively, downstream assay reagents may be chosen with the anticoagulant in mind. Different polymerases have different sensitivities to inhibitors (119). Post-collection sample treatment, for example with NaF/KOx, has been described (119), but additional work is needed in this area before firm recommendations can be made. We note that the Scientific Standardisation Committee of the International Society on Thrombosis and Haemostasis has recommended the trisodium salt of citrate for EV studies [see, e.g. (124–126)].

The necessity of choosing an anticoagulant to fit the specific research question is illustrated by a recent report that the use of calcium chelators promotes rapid association of EV with platelets *in vitro* and thus lowers the apparent count of EV in blood treated with EDTA, ACD or citrate alone (127). This effect was not seen with heparin, prompting the authors of the study to suggest that heparin should be used when accurate EV counts are required (127). However, a previous study concluded that heparin might stimulate the release of EV *in vitro* (128). We recommend that additional research be conducted and published to resolve the important questions of whether and how specific anticoagulants and combinations thereof (122) affect EV release, function and downstream assays such as PCR.

#### Venipuncture and handling

Platelets are susceptible to the activation and release of platelet-derived EV by physical forces associated with the blood draw procedure (129), including contact, pressure and shear forces, so standardization of sampling site, needle gauge and other variables is recommended (130). Use of a 21-gauge needle or larger and avoidance of butterfly systems have been advised for venipuncture to minimize shear forces (131). However, butterfly cannulae (132) and smaller needles (133) have been compared with 21-gauge needles with few reported differences (134). Nevertheless, because different studies have examined different markers of platelet activation and little has been published on the effects of draw procedure on platelet-derived microparticles, standardization is essential to ensure study-to-study comparability. Following venipuncture and first accumulation of blood in the collection tube, the cuff should be removed and the first several millilitres discarded because of the activating effects of pressure (133) and contamination by fibroblasts.

Collected blood should be handled gently and processed rapidly. Tubes may be inverted 8–10 times without shaking for proper mixing with anticoagulant. Tubes should then be stabilized and vertically positioned prior to centrifugation with a recording made of bench-top storage time (135), since the interval between draw and centrifugation to remove cells and platelets may be crucial (136). EV may be stable for the first 30 min after draw (127), but lengthy incubation prior to processing results in increased EV levels (136). Additional studies of this parameter would be illuminating.

#### The circadian system, time of draw and fasting status

Haematologic parameters vary throughout the day (137–139), and slight variations in viscosity have been recognized for several decades (140, 141). Recently, circadian rhythm was found to have a greater effect on platelet activation than 3 stressors including physical exercise (142). Leukocyte trafficking and the presence in circulation of pro- and anti-inflammatory cytokines change throughout the day (143). Large microparticles with specific surface molecules were found to vary with the time of day (144). For these reasons, until there are well-controlled studies designed to explore the effects of diurnal variation on EV, appropriate comparison of results is most easily performed for samples that have been collected at the same time of the circadian day. Additionally, donors with deviation from the normal sleep–wake cycle should be noted. The effects of food intake on EV are not well known, but because lipoproteins carry RNA and food intake affects circulating lipoprotein particle type, number and function (145–147), it may be advisable to draw blood at a defined interval after the last meal. Collection of food intake history could be valuable.

#### Centrifugation and viscosity measurement/adjustment

Blood should be processed quickly and at room temperature to obtain plasma (or serum, as needed). All samples to be studied should be spun at the same speed and with the same rotor type. The viscosity can be assumed to be roughly the same for most blood samples, but any suspected disease-related departures from the norm should be measured (148) and noted since viscosity has been shown to affect EV pelleting efficiency (149). In the setting of elevated sedimentation rate, hyperviscosity IgM syndromes, cryoglobulinaemia and Waldenström's macroglobulinaemia and monoclonal gammopathy of undetermined significance (MGUS) (150–152) consideration should be given to normalization of viscosity by dilution prior to centrifugation (149). (Note: In contrast with whole blood, which is not usually viscosity adjusted prior to removing cells, it is recommended that at least some cell-free biofluid products are diluted with buffer prior to EV isolation; see EV isolation recommendations below.)

#### Paired samples and additional data collection

From each sample of blood to be used for EV isolation and analysis, purification of cells is encouraged to facilitate comparison of vesicular characteristics such as lipid, carbohydrate, nucleic acid and/or protein profiles with those of potential cells of origin. (It should be recognized that cell purification requirements cannot be met if gel-barrier Vacutainer tubes are used at the collection step.) Collection of standard cell and metabolic panels is also recommended to maximize the utility of each sample for comparative analyses. For a more exact quantification, in addition to flow analysis of vesicles, flow cytometry of cells using antibodies to blood and other cells should be performed on the cellular fraction recovered from the body fluid. Since EV in the peripheral blood may be derived from sources other than blood cells (153), characterization of endothelial and circulating tumour cells could also be performed. Obtaining multiple biofluids from the same patient is also desirable, for example, matched CSF and plasma samples.

#### Haemolysis

Haemolysis should be assessed visually and, if necessary, through spectrophotometric and haemoglobin release assays (154). When total exRNA is analyzed, haemolyzed blood products contain higher concentrations of certain RNAs, including miRs-16 and -451 (155). It remains to be determined whether RNAs released in this way will confound the results of evRNA analysis.

#### Removal of platelets

The extent to which platelets are or should be removed from plasma confounds the literature (123). There are protocols for separation of platelet-rich and platelet-poor plasma [PRP and PPP, respectively, see e.g. (156)], but the definition of PRP varies, and truly platelet-free plasma is difficult to obtain. Centrifugation required to remove all platelets would also eliminate large EV. For filtration, forces applied to expedite the process may result in platelet activation or particle fragmentation. Immunoselection likely does not remove all platelets. The tolerance for platelets and platelet-derived EV must be established for each research question. For example, disease-associated mutations (SNPs, indels, rearrangements) may appear from platelet-derived EV (157). Thus, for certain exRNA mutations in biofluids, platelet removal may be preferred, but for others, preference may be given to inclusion of platelet-derived EV. The same protocol should be followed to isolate all samples compared in each study. It may be helpful to determine and report the initial concentration of platelets in each blood sample, and to use markers to track platelet contamination in EV preparations.

## Sample collection and processing from biological fluids other than blood

As noted above, studies of EV in blood products dominate the literature; however, EV are found in and presumably serve important functions in many other biofluids. In this section, we examine several biological fluids and discuss characteristics of these fluids. This section is not a comprehensive catalogue of biofluids or a review of EV research in these fluids, and we do not wish to imply that the biofluids we discuss here – nasal secretions, saliva, urine, breast milk, CSF and the “special case” of cell culture-conditioned medium – are necessarily of greater interest than the many biofluids that are not reviewed. Instead, our purpose is to underline the importance of considering biofluid-specific factors when designing and executing EV studies, from sample collection to EV isolation.

### Saliva

Saliva is a complex fluid that includes glycoproteins, antimicrobial compounds and secreted antibodies. It is produced by 3 major paired glands (the sublingual, submandibular, and parotid) and additional smaller glands lining the oral cavity. Relatively few studies have addressed EV in saliva in disease. However, saliva EV may be markers of oral cancer (158) and the autoimmune disease Sjögrens syndrome (159). EV in saliva have been proposed to participate in immune signalling (51), and uptake of these EV by macrophages has been demonstrated (35). Salivary EV have also been reported to enhance wound healing in a novel evolutionary explanation of the “wound licking” phenomenon (52).

#### Saliva collection

Factors that should be considered in saliva collection include sampling location, time, instructions to participants and collection technique. Since different levels of salivary analytes may be produced by the different glands (160), the location of saliva sampling within the oral cavity may influence results, necessitating standardization within each study and full disclosure of techniques for interstudy comparison purposes. So far, most EV have been analyzed from whole saliva (35, 51, 161). However, EV production and composition might differ between glands, and studies of source-specific saliva, such as the collection of parotid saliva only (162), might be highly important.

Salivary analytes vary diurnally (163), so the time of collection should be standardized. Saliva contents also change with food and drink intake, smoking (164), and in response to stressors including physical activity (165). It has thus been recommended that saliva donors refrain from smoking; from eating or drinking anything except water for 1 h before sample collection; and from strenuous physical exercise prior to collection (166). Because blood contamination could drastically affect apparent saliva content (167), samples should be assessed for the presence of blood. Study participants should not have brushed their teeth within 1 h of collection and should not have undergone dental procedures in the previous days. Any oral disease should be noted. Saliva collection techniques may be separated broadly into stimulated and unstimulated (or passive) sampling (166). For the former, the subject is either given a chemical stimulus or chews on a substance or device to stimulate the release of saliva, which may be drooled or spit into a container or collected with an absorbent material (a “salivette”). Location of collection within the oral cavity is important since the glands differ in their relative contribution to stimulated saliva (168). In passive sampling, the donor empties the mouth of saliva that collects during a defined period of time in the absence of stimulation. How stimulation affects EV production and composition is not currently known, as most studies on saliva EV have been conducted on unstimulated saliva (35, 51, 161). The few studies stimulating saliva production for the analysis of EV have used 0.4–2% citric acid (159, 162).

#### Sample handling and EV isolation

Collected samples should be kept on ice, and consideration should be given to the removal of cells, bacteria and potential food debris prior to EV isolation. This can be done by low-speed centrifugation and the utilization of 0.2-µm filters. It is important to remember that the use of such filters will eliminate many large EV. Vacuum filter systems may be helpful to use instead of syringe filters, as clogging of these smaller filters are common when working with saliva. As for other viscous body fluids, dilution with PBS is recommended. Isolation of saliva EV has been performed by differential centrifugation (35, 158, 161) and gel filtration (51, 169). We recommend that additional studies be performed, for example, to determine whether collection of saliva by absorbent techniques is compatible with EV isolation.

### Urine

Urine is increasingly studied as it can be obtained non-invasively in large volumes and contains EV with diagnostic potential (170) for renal, bladder, urethral and prostate diseases including cancers (171, 172). RNA is protected by urinary EV to a greater degree than by cells in urine (173), and the EV themselves are remarkably stable (174).

#### Collection and study design

If voided urine is collected, attention should be paid to potential bacterial contaminants. It is advised to consider characteristics of “freshly” voided urine as distinct from large residues stored in the bladder. Ease of collection means that normative controls could be selected from relatives accompanying the patient (preferably sex- and/or age-matched individuals). Care must be taken in situations where the patient but not the control donor has been advised to fast or follow other dietary changes due to health procedures.

#### EV isolation

Several groups have reported that rapid nanomembrane-based concentration of urinary EV is a reliable alternative to lengthy ultracentrifugation procedures (170, 173, 175). Expedited processing options could speed up the introduction of urine-based diagnostics (173), as could the utility of frozen urine for EV isolation [as referenced in (173)].

A characteristic of urine, which may in some cases complicate EV isolation, is the presence of the Tamm-Horsfall protein (THP, also referred to as uromodulin), a major protein component of urine (176). Polymeric THP may entrap large amounts of EV, which may thus pellet at low speed (177). Addition of the reducing agent dithiothreitol (DTT) releases EV from the THP trap. However, upon addition of DTT to urine samples, monomeric THP will be still present in EV that are pelleted at high speed (178). The zwitterionic detergent CHAPS (3-[(3-cholamidopropyl)dimethylammonio]-1-propanesulfonate) has been proposed recently as an alternative to DTT in removing THP entanglement, while retaining vesicle morphology and protein activity (179). Use of sucrose cushions or gradients with heavy water (but not regular water) has also resulted in successful separation of EV from THP complexes (67, 177).

### Nasal secretions

Pathological changes during airway diseases and inflammation can be monitored by measuring cells and soluble mediators in nasal secretions and by observing epithelial remodelling in nasal biopsies (180). EV can be isolated from nasal secretions (53) and may have altered function(s) and cargo during respiratory disease. Understanding nasal EV is of particular importance since therapeutic vesicles could be transported to the brain through the olfactory system (181).

#### Sample collection

Although the influence of sampling technique on yield, purity and stability of EV is not currently known, nose blowing, nasal lavage, suction, swabs and spray and absorption techniques (182) may result in differences in cell count, concentrations of soluble proteins and level of caused irritation to the epithelium (182–184). Sampling-induced epithelial inflammation and damage should be kept to a minimum. Since inhaled particles vary by season, climate and degree of urbanization (185–187), baseline samples or pools of samples from several collections could overcome within-subject variations (53). As allergens induce inflammation in the nasal epithelium, atopic subjects are likely to be even more sensitive to seasonal changes (188). Same-season collection and controlling for atopic status, occupational exposure and degree of urbanization are recommended. For collection of multiple samples, protein studies indicate that between-collection interval should be considered and standardized (182). Studies are needed to determine if loss of yield through rapid succession sampling also applies to EV, but the epithelium and mucus layer may need time to recover between sampling.

#### Recovery

After instilling 2.5–5 ml of saline to each nostril, recovery rate is approximately 80% but can vary between subjects (range: 65–90%) (180). Loss can be limited by having the subject close the soft palate and train in the NLF procedure (182). Keeping the mouth closed during collection ensures that that fluid collected is from the nose, and does not include saliva. It is possible that the yield of nasal luminal vesicles can be improved by increasing the volume of lavage fluid.

#### Age and gender

Although knowledge of the production and recovery of EV in nasal secretions remains limited, it is probably important to study age- and gender-matched subjects. Pro-inflammatory molecules and second messenger in nasal secretions can differ with gender (185, 189), and protein concentration in nasal mucus can decrease with age (189).

#### Handling of the collected nasal secretion

Little is known about the best handling of collected samples for EV studies. However, cells should always be removed by a gentle centrifugation before the samples are stored in a freezer. The pelleted cells may be examined for number of inflammatory cells, as well as contamination of saliva cells and blood cells to establish the condition of the collected sample.

### Breast milk

Breast milk provides not only the required nutrients for neonates but is also thought to protect against a variety of health problems in children including necrotizing enterocolitis, childhood obesity, severe infections, as well as reducing the risk of cardiovascular disease and cancer in later life (190, 191). Breast milk is a very complex fluid consisting of proteins, carbohydrates, lipids, cells and other biologically important components. Human breast milk contains non-nutritional bioactive components, such as maternal cells and antibodies, chemical mediators, vitamins and enzymes (192). Recent studies have reported the presence of EV in human breast milk (35, 37, 193) and cow's milk (194–196), and several types of RNA were described to be associated with these vesicles (35, 193, 195). Various protocols of differential (ultra)centrifugation and density gradient ultracentrifugation were used in these studies for isolation of EV. Milk samples had often been stored at low temperatures before isolation of EV. This may have affected the milk EV composition. Although EV in milk were shown to have immune modulatory effects (37), the role of milk EV in neonates awaits clarification. Furthermore, EV in milk are being explored as biomarkers to monitor the development of (immune-related) diseases such as allergies.

The selection of patient groups and normative controls should be carefully considered when studying breast milk because of the large number of factors that influence the composition of milk. These factors include the stage of lactation, parity, volume of milk production, infant feeding, maternal diet and energy status, maternal health, illness, and stress (197). For example, the composition of colostrum (1–5 days), transitional milk (6–15 days) and mature milk differs largely with regard to the concentration of fat, proteins, vitamins and antibodies (198, 199). Furthermore, it should be considered that, during active phasing out of breastfeeding, apoptosis events causing mammary involution (200) may lead to increased numbers of EV derived from apoptotic cells in the milk EV populations.

Isolation of EV from milk is complicated by the high lipid content of milk. Lipids (mainly triacylglycerols) are released in milk as fat globules (MFGs) by mammary epithelial cells. These MFGs are droplets of lipids surrounded by a complex phospholipid trilayer containing proteins and glycoproteins (201), and thus are a type of EV. MFGs are largely heterogeneous in size, and their buoyant densities are different from those of other EV (201). Because of their vesicular nature and high abundance in milk, however, MFGs may be co-isolated with other EV populations present in milk.

Several lines of evidence indicate that the method of milk sample collection and storage affects the composition of milk, and may therefore also influence the EV content of milk. The method of milk expression (manual versus electric pump) was shown to influence the fat content of milk and contaminations derived from the skin (202). Since the composition of fore and hind milk has been shown to be largely different (203), milk samples for research mostly consist of equal volume mixtures of these two milk types or complete milk of one feed. Various studies have addressed the influence of low-temperature storage on breast milk composition [reviewed in (202)]. The type of storage container (glass, polyethylene or polypropylene) has been shown to influence cell content (202) and could therefore affect the EV content. Freezing of milk, as is widely performed for milk banking, reduces cell number (204) and dying cells lead to contamination of the milk EV population (E. N. Nolte-‘t Hoen, personal communication). Freezing milk also leads to loss of bile salt dependency of the bile-salt-dependant lipases (205), and increased lipase activity after thawing may affect cell and EV integrity. Additionally, freezing breaks the emulsion between MFG and the aqueous fraction, which may lead to changes in EV isolation efficiency and purity. Storage of milk at higher temperatures can also affect milk composition. Incubation at 15–35°C was shown to lead to a decrease in pH within hours and rapid lipolysis (within 1 h). Importantly, not only freezing, but also storage at 0–4°C leads to a reduction in the number of milk cells [204 and E. N. Nolte-‘t Hoen, personal communication]. We conclude that additional EV-specific studies would be helpful to establish the effects of collection and storage methods on milk-derived EV and their content.

### CSF

CSF bathes the central nervous system and is continually filtered into the blood. Although CSF typically contains much lower concentrations of EV than other biofluids including blood, the presence of EV in CSF has been reported based on methods including transmission electron microscopy (TEM) and laser correlation spectroscopy (54, 206–208). Given their potential as reservoirs for biomarker discovery in neurological disorders, different research groups have profiled the EV-associated proteome and lipidome composition associated with EV (54, 207, 208). The identification of protein structure and modifications within CSF EV may represent a key component in deciphering RNA–protein interaction within disease-associated modifications that may be important elements in induced neurodegeneration. There is also interest in identifying the key players – proteins and RNAs – in unconventional EV release mechanisms prominent in neurodegenerative disease pathogenesis (209).

Comprehensive guidelines have been drawn up for CSF biobanking (210). CSF is collected from lumbar regions L2/3 or L3/4 according to consent and approved medical protocol (210). Investigators should be vigilant for contamination of CSF samples with blood. For EV concentration, samples are often subjected to differential centrifugation (54, 207, 208). As for isolation of EV from other biofluids, standardization of techniques has not yet been achieved and will require comparative studies.

For CSF, it may be particularly problematic to obtain healthy control samples, although such samples appear to be available commercially (211). Appropriate controls may be obtained from patients suffering from disorders other than that investigated in each respective study (e.g. affective disorders, peripheral neuropathy, persistent headache, normal pressure hydrocephalus) (212, 213) or those undergoing intra or epidural procedures.

### Cultured cell-conditioned medium

EV and evRNAs isolated from biofluids necessarily have a mixed cellular origin. In some cases, it may be interesting to analyze specifically vesicles and RNA recovered from a single cell type. This is easily achieved by collecting conditioned medium from cultured cells. EV are generally purified from medium conditioned by cells for 24–48 h, but shorter conditioning times have been used to analyze specific release of EV-associated markers after a specific stimulus: for instance, surface receptor-mediated stimulation (214, 215) or fMLP, PMA or calcium ionophore treatment for 15–60 min (1, 216, 217). It is important to keep in mind, however, that such acute stimulations may lead to the release of EV of different intracellular origin than those secreted spontaneously, and no extensive side-by-side comparisons of steady-state and induced EV have been performed to our knowledge so far. Even in this simplified situation, precautions to avoid artifactual EV are indicated.

#### Serum-derived EV

In most cases, cells are cultured *in vitro* in the presence of serum. Whether this is human serum or foetal calf serum (FCS), it contains EV and RNAs. To avoid contamination of cell-derived vesicles by serum EV, 2 solutions have been proposed (70). The first is to eliminate serum-derived vesicles before cell culture by performing an overnight ultracentrifugation at 100,000×*g*. To allow efficient elimination of vesicles, and due to the viscosity of serum, it is mandatory to centrifuge serum diluted to at most 20% in the culture medium. To make sure that the medium has been properly depleted from vesicles, quantification by Western blot using antibodies recognizing bovine proteins classically found in EV, such as transferrin receptor or flottilin-1, can be performed. The second solution is to culture cells in serum-free medium. This is possible, for instance, for the culture of primary neurons, which are classically cultured in a defined medium with growth factors. For cells that are normally grown with serum (e.g. tumour cell lines), but that can survive without serum for a few days, many laboratories simply change the medium to serum-free medium for the conditioning time. Such an abrupt switch to nutrient-poor medium, however, will unavoidably induce a stress response, most probably leading to secretion of EV of different composition and/or intracellular origin. Here again, however, no specific side-by-side comparisons have been performed yet, and it would be advisable to perform such comparisons for each type of cells analyzed. Finally, some commercially available serum-free media for long-term growth of some cell lines, such as AIM-V, give rise to a pellet containing proteins (as quantified by microBCA) and vesicles or protein aggregates (as quantified by Nanoparticle tracking analysis; C. Théry, personal observations). Thus, these media may not entirely circumvent the problem of contamination by non-cell-derived vesicles.

#### Cell death

Whatever the culture conditions, it is absolutely necessary to quantify the percentage of dead cells present in the culture at the end of the conditioning time. Indeed, dying cells release vesicles of various sizes, and eventually break into cell fragments, which can in turn fragment into smaller vesicles upon ultracentrifugation or filtration. Presence of abundant dead or dying cells will thus lead to contamination of live cell-derived EV by dead-cell-derived vesicles, and current purification protocols will not separate these different vesicles. Soluble materials released from dying cells may also adhere non-specifically to lipid vesicles, and thus change their protein and/or nucleic acid composition. For these reasons, one might arbitrarily specify 5% as the maximum acceptable cell death percentage in culture to provide reasonably pure EV released by live cells.

#### Microbial contamination

Finally, microbes may contaminate cell cultures. If conditioned medium is subjected to high-speed pelleting, any microbial contamination is expected to sit in the pellet. Authors should be aware of this risk, and first confirm the Mycoplasma-free status of cells used in their experiments. Similarly, if cells are productively infected with viruses, they will co-purify with small EV upon ultracentrifugation. One could even imagine that endogenous retroviruses, normally present in mammalian genomes, may recombine with genomes of retroviral expression vectors, and lead to production of new viruses, although this has never been strictly demonstrated.

## EV isolation

### Method-independent considerations

#### Input volumes and dilution

Optimal input volume for EV isolation will likely need to be determined empirically for each biological fluid and isolation method. In the case of RNA purification from EV or fluids, it has been suggested by some (119) that larger amounts of starting material do not necessarily result in larger amounts of recovered RNA, perhaps because of clogging or saturation of RNA purification columns or co-purification of PCR inhibitors. Dilution of biological fluids with PBS may enhance the recovery of EV by altering the viscosity of the respective fluid (149), a principle that was noted as early as 1967 for EV that was then described as “platelet dust” (218). Théry and colleagues recommended dilution of plasma, ascites, BAL and other fluids with an equal volume of PBS prior to centrifugation (70). Others have reported dilution by factors of 3- to 10-fold (13, 108, 219). However, the need for and utility of dilution is viscosity dependent and may not be necessary for every biofluid.

#### Storage and handling of collected fluid before EV isolation

It may be advisable to proceed to vesicle isolation immediately after collecting the biofluid or cell-conditioned medium that is to be analyzed (136). In some cases, however, fluid storage before EV purification may be convenient, for instance to allow simultaneous processing of samples from different patients or sources. One might also wish to examine previously biobanked patient samples. At a minimum, cells and platelets should be removed from the fluid prior to storage. Beyond this elementary consideration, little consensus exists, and reports of well-controlled experiments are needed to reveal the consequences of fluid storage temperature (4°, −20°, −80° or −160°C) and other handling and storage parameters for the recovery and phenotype of EV.

#### Storage and handling of isolated EV

Storage vessels, buffer and conditions may each affect the outcome of EV experiments. Siliconized vessels are recommended for storage to prevent adherence of EV to surfaces. At the least, vessels should be tested to ensure that EV are not partially or completely lost to surfaces. PBS is the standard choice for resuspension (70), and the current consensus seems to support storage at −80°C. The effect of additives including dilute, highly purified protein carriers, awaits detailed investigation. As for any biological material, it makes sense to minimize freeze/thaw cycles, although evidence was presented at the October, 2012 ISEV workshop that small vesicles are relatively insensitive to freeze/thaw cycles and may even resist bursting in a hypotonic environment. Resistance to freeze/thaw has also been reported in the literature (127, 220). Nevertheless, the best practice is to compare frozen with frozen and fresh with fresh, and to record any and all freeze/thaw cycles to which each sample has been subjected. It should also be clearly indicated whether vesicles were isolated from fresh or frozen samples. Finally, samples should be both frozen and thawed rapidly for maximal preservation of morphology and function. However, some participants at the ISEV research workshop reported loss of function following freezing and advocated use of vesicles stored at temperatures just above freezing. Additional studies are needed to identify storage and handling methods that allow optimal retention of function.

### Methods

EV isolation may be achieved by a variety of methods, including ultracentrifugation, filtration, immunoaffinity isolation and microfluidics techniques. The invention of the ultracentrifuge by Svedberg around 1920 (221) led to the extensive development of separation techniques that were applied to the study of enveloped viruses – an interesting subset of EV – in the mid-20th century. Ultrafiltration methods were applied even earlier (222), while immunoaffinity purification of viruses was performed in the 1970s [see, e.g. (223)]. It is beyond the scope of this article to present this important historical backdrop to current EV research. Instead, we will describe the ground-breaking application and modification of these methods to investigations of non-viral EV (64, 70). These methods are not necessarily mutually exclusive, and combinations may be desirable (70, 224–226). Choice of method(s) should be guided by the required degree of EV purity and concentration.

#### Centrifugation

To date, most published studies of EV from biofluids or cell culture have employed differential centrifugation with or without size filtration to concentrate and partially purify EV (64, 70). After depletion of cells, platelets and large apoptotic bodies by 1 or more low-speed centrifugation steps and/or size exclusion (e.g. 800-nm filter), larger EV (variously labelled microvesicles/microparticles/ectosomes) are pelleted. Forces commonly reported for this step are in the 10,000–20,000×*g* range. Smaller EV are then pelleted at high speed (100,000–120,000×*g*). Such stepped ultracentrifugation procedures cannot achieve absolute separation by size because sedimentation also depends on the density or “cargo” of a particle and the distance a particle travels. Some small EV near the bottom of the tube will pellet in the large vesicle pellet at low speed (226), while some larger particles near the top of the tube may pellet only with the high-speed spin. Aggregation of EV, a common occurrence, also interferes with separation by individual vesicle size. Similarly, the pellet from a high-speed spin will contain extravesicular proteins complexes/aggregates, lipoprotein particles, and other contaminants. Resuspending and recentrifuging each pellet in PBS may aid in removing some of these impurities, albeit at the risk of reducing the yield of EV, but absolute separation is impossible by this method alone. For this reason, protein measurements of EV-containing pellets are inadequate to determine recovery of EV [we also note that lipids are known to interfere with detergent-compatible protein assays (227)].

To remove contaminating non-vesicular particles, density gradients add stringency by separating particles of different density. By allowing EV to float upward into an overlaid sucrose gradient, as described in the original studies of EV (64, 70), one can efficiently separate contaminating protein and/or protein–RNA aggregates, which will remain at the bottom of the tube, from lipid-containing vesicles. In addition, this procedure allows separation of vesicles according to their density, classically reported between 1.1 and 1.19 g/ml. Of note, 2 studies published in 2012 showed that some vesicles take between 62 and 90 h (228, 229) to reach equilibrium density, that is much longer than the traditional 16-h centrifugation time (70). Thus, performing “buoyant velocity separation” as proposed by Palma et al. – introducing variable lengths of centrifugation of the sucrose gradient – may allow more efficient separation of different vesicles. Finally, another commonly used approach involves loading the pellet on top of a sucrose gradient (33, 41, 42), a sucrose cushion (171, 224) or iodixanol (71) before centrifugation for a few hours to overnight. If centrifugation is too short and/or the tube is too long, though, contaminating aggregates may not reach the bottom of the tube and thus end up in the same fractions as EV. Since different classes of vesicles may have overlapping densities, however, even density-based procedures may achieve enrichment rather than true isolation. Where reconstitution of biological activity is required, the effects of ultracentrifugation parameters, including the influence of sucrose and changing the osmotic environment, might be carefully assessed.

When reporting results of centrifugation experiments, it is essential to specify all variables of the centrifugation procedure (230) to ensure the possibility of reproduction and study-to-study comparison. Physical separation depends not only on the *g* force applied, but also on the rotor type (fixed angle, swinging bucket), pelleting efficiency (rotor *k*-factor), sample viscosity, and gradient characteristics. Several participants at the ISEV 2012 workshop encouraged the exclusive use of swinging bucket rotors during ultracentrifugation. In comparison with a swinging-bucket rotor, a fixed-angle rotor deposits material against the wall of the centrifuge tube and gradually forces this material down the wall to the bottom of the tube. There are competing views on whether ultracentrifugation in general damages EV (220, 226). This process is thought by some to be physically damaging to viruses; effects on non-viral particles are largely unknown, but aggregation may occur. Well-controlled comparisons are needed to compare size, percentage of damaged vesicle, soluble protein contamination and RNA profiles associated with different methods.

#### Size exclusion

Separation of EV based on the size and passage through physical barriers may be achieved through the use of filters or chromatography. In the former, particles larger than the desired size may be excluded, for example by a filter with a 0.8-µm pore size, or particles smaller than the desired size range may be removed, while the target population is retained on the filter. Column chromatography allows for sequential elution of EV size fractions from a single column. Filter-based methods will not enrich EV populations unless low-molecular-weight filters (e.g. Centricon units) are used for concentration. (However, little is known about the effects of this concentration on EV and the extent to which target EV populations may adhere to the filter and thus be lost to analysis.) For this and other reasons, size exclusion methods are often combined with ultracentrifugation or other techniques. Filers of 0.8 µm are often used to remove large cell fragments and platelets prior to EV isolation (63), while 0.2 µm filters may be used when smaller EV are desired (70). Participants at the 2012 ISEV workshop noted that forcing particles through filter pores may cause deformation and breakup of large vesicles or platelets, thereby potentially skewing results. To prevent this, size exclusion could be performed by gravity (63) or, if this is impractical because of time considerations, by the smallest possible applied force. It is also advisable to check that filters do not release contaminating debris/particles that may interfere with downstream applications.

#### Immunoaffinity isolation

The presence of characteristic surface proteins on certain EV classes is the basis for immunoaffinity isolation (70, 231–233), in which antibodies to surface proteins are used to select desired EV populations positively (immuno-enrichment) or to trap unwanted EV populations (negative selection or immuno-depletion). Antibodies are associated with beads or other matrices (234–236) through covalent or high-affinity interactions, facilitating physical separation by low-speed centrifugation or magnetic techniques. Although commonly used (237), sometimes in combination with other methods (225, 238), immunologic approaches have diverse input requirements, antibody/antigen combinations and attachment/isolation systems. As an example of an immunologic approach to vesicle isolation, HIV-1 virions have been reported to exclude the cellular membrane tyrosine phosphatase CD45 (leukocyte common antigen), allowing purification of virions by negative selection from lymphocyte EV (239, 240). Depending on the approach, this method can be used to purify and enrich EV.

Immunologic techniques have the potential for high specificity (241, 242), an important consideration in the characterization of unique EV populations. They may also be used to select for EV with the correct membrane orientation, that is vesicles with a cytosolic-outwards orientation cannot bind antibodies raised to exoplasmic features. However, selectivity also results in concomitantly lower yields than methods that rely on physical properties, not least because some markers are possibly not represented or recognized on all vesicles within a given class (70). For reporting, it is recommended to assess not only the presence of selected markers, but also the absence of contaminants, and to include appropriate isotype controls.

#### Polymeric precipitation

With the goal of enriching EV, commercially available polymeric precipitation mixtures are incubated with biofluids, typically overnight, and low-speed ultracentrifugation is used to collect precipitate that is said to contain EV or, specifically, “exosomes”. These methods, using reagents such as ExoQuick from System Biosciences, are technically facile and require little hands-on time (243), but there may be concerns about the purity of the resulting product. In one study of small EV isolation from ascites fluid, for example, it was concluded that polymer-based precipitation was superior than size exclusion, immunoaffinity separation and ultracentrifugation (244). However, only recovered RNA and protein purity and quantity were assessed in this study, and the results would thus be consistent with greater recovery of RNA or protein through inclusion of impurities. At the 2012 ISEV workshop in New York City, participants discussed an apparent tendency of polymer-based precipitation methods to include numerous non-EV contaminants such as lipoproteins. In a publication that assessed exosome morphology following ultracentrifugation, precipitation and combination methods, it was also reported that density gradient ultracentrifugation was preferable even to a precipitation/UC hybrid method for recovery of high-quality small EV (196). Polymer-based precipitation may produce high yield of RNA and may be appropriate in the case of fluids that are thought to have enriched small EV fractions (245), but rigorous assessment of contaminating particle fractions is recommended when this method is employed.

#### Microfluidics

In 2010, Chen, et al. demonstrated rapid recovery of small EV from both serum and conditioned culture medium with a microfluidic device containing antibody-coated surfaces (238). Other micro- and nanofluidics methods separate nanoparticles based on physical properties alone (246, 247). The application of these methods to biological fluids has not yet been described extensively.

## Quantitative and qualitative assessment of EV

Various optical and non-optical methods have been developed or adapted for the assessment of EV quantity, size and features such as the presence of specific surface markers (248). Optimization and standardization of protocols remain an important task. The following is an overview of a selection of available techniques, and some methods that are not covered here may play an important role in on-going and future EV studies.

### Techniques

#### Electron microscopy

Electron microscopy (EM) techniques are well established and have proven very useful in EV research (64), providing direct evidence for the presence of vesicular structures. The use of heavy metal stains such as osmium tetroxide and uranyl acetate in TEM enables recognition of membrane-surrounded vesicles, and contrast is enhanced by embedding in methylcellulose. In most cases, concentrated EV suspensions are applied to grids and fixed, for example with paraformaldehyde. As an alternative method, EV pellets at the bottom of a centrifuge tube are fixed, and ultrathin cross sections are analyzed by TEM (63). While using suspensions is simpler, sectioning pellets may be more informative by showing substantially higher numbers of EV in the EM field and avoiding the possibility that certain types of suspended EV do not adhere to the grid. TEM is frequently combined with the use of immunoglobulins coupled with nanogold particles that detect specific EV features (immuno-EM). Further EM tools to investigate EV include scanning EM (220, 249) and cryo-EM. The latter enables the analysis of EV in frozen samples with the advantage of avoiding the effects of dehydration and chemical fixatives (250). For example, cryo-EM results indicate that the cup-shaped appearance of “exosomes” as reported by TEM is an artifact of preparation (251). EM is valuable for assessments of morphology, size and the presence of markers (by immuno-EM). The technique is of limited use for concentration measurements.

#### Atomic force microscopy

In atomic force microscopy (AFM) (252, 253), a mechanical cantilever is passed over a surface, with deflections indicating the presence of surface structures. With the possibility of sub-nanometre resolution, AFM is particularly suited to assessments of EV morphology. AFM has been applied to the study of EV in numerous publications [e.g. (63, 254, 255)].

#### Optical single particle tracking

Nanoparticle tracking analysis (NTA) is the commercial name of an optical particle tracking method for obtaining concentration and size distribution of EV populations (256–258). A laser beam is scattered by particles in the sample, and the mean velocity of each particle is calculated by the Stokes–Einstein equation on the basis of Brownian motion recorded by a CCD camera. For accurate quantification of number and size of heterogeneous populations of vesicles, the procedure requires accurate optimization of camera and analysis settings, and separate measurements with different settings may be needed to obtain accurate readings for EV subsets in heterogeneous mixtures. A technical article published recently in *Journal of Extracellular Vesicles* should help with standardization of these procedures (259). Although standard light scatter NTA does not detect biochemical composition or cellular origin of vesicles, analysis of fluorescently labelled vesicles is also feasible (256, 260). This, too, requires optimization, and as yet is not used routinely. Of note, prior to NTA analysis of biofluid EV, for example from blood, isolation of vesicles by centrifugation or other means is necessary in order to remove lipoprotein particles, protein complexes and other particles that may have similar size and outnumber EV in the blood. Indeed, when using NTA in light scatter mode, one might keep in mind that large protein aggregates will not be distinguished from vesicles with similar Brownian motion, and thus even after partial purification, the particle concentrations calculated by this technique include a mixture of EV and non-vesicular structures. As for flow cytometry, it could be useful to compare samples treated or not with detergents to eliminate vesicles (see “Flow cytometry” section), but the influence of detergent on NTA-based analysis requires further investigation.

#### Resistive pulse sensing

Resistive pulse sensing, commercialized as the IZON qNano technique, is a novel alternative to NTA for concentration and size distribution measurements. This technique detects individual EV by transient decrease of an ionic current caused by the transport of a vesicle through a nanopore in a membrane (261–263). Polydisperse systems, such as heterogeneous vesicle populations, often require the combination of results obtained using more than 1 nanopore membrane (each of which is used for detecting a limited size range of particles).

#### Dynamic light scattering

Dynamic light scattering (DLS) calculates the average size of relatively monodisperse populations of isolated particles (220, 257, 258). DLS has also been used for analysis of EV (226, 264). However, for the analysis of heterogeneous EV populations, this technique is less suited, and results may vary depending on analysis software.

#### Flow cytometry

Flow cytometry is a powerful method for both the qualitative and quantitative characterization of cells and smaller particles, including EV. For single vesicle-based flow cytometry, conventional flow cytometers have a lower detection threshold around 500 nm and are suited only for analysis of large EV. Introduction of the Megamix bead gating strategy was an important step towards standardization of flow cytometric assessment of larger EV (265, 266). Newer instruments such as Gallios (Beckman Coulter), BD-Influx (Becton Dickinson) and Apogee (Apogee Flow Systems) enable discrimination of particles as small as 200 nm (268) or even 100 nm in diameter (266, 268, 269). The issue of appropriate size standards, that is polystyrene beads with high refractive index, silica particles with slightly lower refractive index, or other materials, has been the subject of some discussion (270). Light scatter-based flow cytometry detection of EV may be confounded by protein aggregates, including clumps of fluorescently labelled detection antibodies (271). To circumvent this problem, differential detergent lysis has been suggested to distinguish EV from protein aggregates during flow cytometry (272). Investigators who use this approach may wish to demonstrate carefully that EV are appropriately eliminated, since some lipid domains of EV are relatively insensitive to weaker detergents (233, 273). Calcium-phosphate microprecipitates were also shown to result in EV-mimicking signals by flow cytometry (274).

Recently, a protocol was published for high-resolution flow cytometry detection of fluorescent EV subjected to flotation on sucrose, and using custom-made settings of the BD-Influx (Becton Dickinson) (269). This method overcomes the interference by protein aggregates or calcium-phosphate microprecipitates. However, analysis of small vesicles by flow cytometry requires very careful controls performed using identical settings, including display of the plots and quantifications collected and calculated from the diluent alone, and from standard beads of a similar size and preferably with a comparable refractive index to that of the analyzed vesicles. Furthermore, serial sample dilutions should be analyzed to define the proper dilutions at which “swarm” signals generated by multiple vesicles and detected as a single event are minimized or excluded [(275) and M.H. Wauben, personal communication].

To allow easy analysis of EV by flow cytometry, it is possible to couple EV to micrometre-sized latex beads (70). In this way, the relative presence of various surface proteins can be determined, but this method will neither allow EV quantification nor discriminate between different vesicle subsets.

#### Western blot

Conventional Western blotting may be used to demonstrate the presence of proteins reportedly associated with EV or EV subgroups in preparations that include EV, but is not suitable for determining EV quantity. Markers might include tetraspanins (CD9, CD63, CD81, CD82), MHC molecules, or milk-fat globule-EGF-Factor VIII (MFGE8, or lactadherin), and cytosolic proteins such as certain stress proteins, Tsg101, Alix, or cytoskeletal proteins (e.g. actin, tubulin). Purified vesicle preparations or pellets of fractions of sucrose density gradient ultracentrifugation may be treated with buffered solutions containing lysis reagents, denaturants and protease inhibitors or directly mixed with loading buffer. Proteins may then be separated by SDS-PAGE. It is important to emphasize that this technique on its own cannot identify whether detected proteins are from EV, and, as discussed below, that the presence or enrichment of an EV marker does not mean that contaminants are absent. Nevertheless, the Western blot is a useful tool for detecting proteins present in purified EV preparations.

### Contaminants of vesicle preparations

In addition to EV contaminants (that is, EV other than those of the desired class), lipoproteins, microbes, microsomes, chromatin and protein aggregates may co-purify in vesicle preparations and confound analysis. Therefore, caution is warranted when describing a given vesicle preparation.

#### Diverse populations of vesicles in preparations

Biological systems contain diverse populations of EV. Of note, current evidence suggests that the repertoire of vesicles secreted by cells is dynamic and varies from cell to cell. In spite of recent efforts to purify a single type of vesicles (such as exosomes as defined by different research groups), preparations usually contain more than 1 population. Even preparations isolated by sucrose gradient ultracentrifugation have been shown to contain diverse types of vesicles (276–278). Evaluation and re-evaluation of putative markers of specific vesicle types is in progress, and additional efforts are needed. Even in the case of markers with strong evidence for EV subtype-specificity, the presence of such markers does not rule out that other types of vesicles are present in a preparation simultaneously. Not only must the desired population be confirmed as present; contaminants must be demonstrated to be absent.

#### Lipoprotein particles

When vesicles are isolated by high-speed ultracentrifugation, most large lipoproteins will be excluded from the pellet by their buoyancy. However, some particles – in particular, high-density lipoprotein, HDL – may co-purify with EV. Although HDL particles are approximately 10 nm in diameter, and thus are substantially smaller than EV, their density overlaps that of EV (15). Direct interactions are also a possibility. Even EV preparations isolated by sucrose gradient ultracentrifugation (1.13–1.19 g/ml) may contain contamination by HDL. How – and if – to separate HDL and other contaminants from the target vesicle population(s) depends on the goals of each project. Given that HDL has been shown recently to transport RNAs (15, 16), attempts should be made to discriminate extra-EV (e.g. HDL-associated) and EV-associated RNA in blood plasma samples. Direct pelleting of small EV and HDL at 100,000 and 300,000×*g*, respectively may discriminate between the 2 types of particles (15).

#### Microbes

The size ranges of EV overlap those of viruses and bacteria (125), and any EV preparation is potentially subject to microbial contamination. EV preparations may contain virions or virus-like particles, including those of endogenous retroviral origin. Use of Optiprep (iodixanol) gradients has been shown to separate virions from small EV (71). Most bacteria, in contrast, are usually pelleted at low speed during differential centrifugation (see the “Cell culture-conditioned media”).

#### Microsomes

Disrupted membrane fragments released by eukaryotic cells undergoing necrosis may re-form vesicular artifacts referred to as microsomes. Contributing membranes include but are not limited to endoplasmic reticulum. Microsomes may be smooth or rough (the latter carry numerous membrane-bound ribosomes). Microsomes pelleted at high speed (100,000×*g*) are potential sources of ribosomal RNA in EV preparations. The presence of endoplasmic reticulum proteins (such as calnexin) may be an indicator of microsomal contamination. As mentioned above, immuno-isolation techniques may aid in selecting non-microsomal vesicles.

#### DNA and other products of necrosis

Organelles and macromolecules including DNA may be released by necrotic cells. DNA/chromatin networks known as “neutrophil extracellular traps” (NETS) are also released as an antimicrobial defence mechanism (279, 280). DNA, on its own or associated with chromatin proteins, may bind to EV, creating aggregates with greatly altered buoyant density. Because these aggregates would pellet at low speed, DNA contamination may result in reduced yields of vesicles purified with differential centrifugation and ultracentrifugation. DNase digestion or gDNA eliminator columns may be necessary to remove DNA contamination of EV samples.

#### Protein aggregates

Protein aggregates may share biophysical parameters (including sedimentation properties) with EV (63, 281). Protein aggregates (such as immune complexes) may pellet along with larger EV at low speed, and are possible contaminants of extracellular preparations isolated from biological fluids. As stated before, segregation of EV from protein aggregates can be achieved by gradient density ultracentrifugation.

## Expectations and proposals

In this position paper, we have summarized discussions that took place at and following the ISEV research workshop in New York City in October, 2012. There exists surprisingly little consensus within the research community on a variety of technical and biological questions. As a result, scientific controversies may simply reflect competitive isolation or analysis strategies. In addition to reviewing issues of sample design, collection, processing; EV isolation; and EV analysis; we also issue several recommendations (Table II). An immediate need is for normative samples from the general healthy population. To ensure adequate supply, multiple pools of individual samples could be produced. These sample pools would be held by one or more central repositories and distributed to researchers on request. In future, it may be desirable to expand the types of stored sample to include those from groups with sedentary versus active lifestyle, high- or low-calorie diet, young and old, male and female, among other characteristics, to provide better matches for disease studies. Similarly crucial is the development of marker-containing and differently sized artificial biomimicks of EV. These vesicles are needed as standards and would be spiked into biological samples to assess isolation efficiency and thus furnish technical normalization. An artificial standard, for example, loaded with a specific RNA sequence, would also allow independent validation of results. Reliable standards could demonstrate that a particular isolation method retains function – RNA transfer capacity – of isolated EV. These standards, perhaps along with calibration standards for the various EV quantitation and sizing technologies, could be made available commercially or distributed through a repository to consortium members.

Of course, strides forward in this field will be possible only with the support of funding agencies such as the European Community and the National Institutes of Health (NIH) of the United States. We applaud the European Metrology Research Program (EMRP) project MetVes (EMRP-MetVes, http://www.metves.eu), designed for metrological characterization of EV from body fluids and the European Cooperation in Science and Technology (COST) program “Microvesicles and Exosomes in Health and Disease (ME-HAD)” (http://www.cost.eu/domains_actions/bmbs/Actions/BM1202). We are also pleased by the NIH Common Fund initiative for exRNA (http://commonfund.nih.gov/exrna/), in which funds have been dedicated to projects involving discovery and validation of exRNA biomarkers of disease diagnosis, prognosis and response to therapy; development of novel exRNA-based therapeutics; and examination of the normal distribution and function of exRNA in various body fluids. We encourage further development of funding opportunities to resolve the many outstanding technical and biological questions of EV and evRNA research. Finally, we anticipate and look forward to progress in the coming years towards this goal.
